# Correlation of Reactive Oxygen Species Levels with Resveratrol Sensitivities of Anaplastic Thyroid Cancer Cells

**DOI:** 10.1155/2018/6235417

**Published:** 2018-07-10

**Authors:** Xu Zheng, Bin Jia, Xiao-Ting Tian, Xue Song, Mo-Li Wu, Qing-You Kong, Hong Li, Jia Liu

**Affiliations:** Liaoning Laboratory of Cancer Genetics and Epigenetics and Department of Cell Biology, College of Basic Medical Sciences, Dalian Medical University, Dalian 116044, China

## Abstract

Anaplastic thyroid carcinoma (ATC) is the most lethal thyroid malignancy without a reliable therapeutic agent. Resveratrol possesses cancer-suppressive effects, while its effect(s) on ATC cells remains unknown. Because oxidative damage caused by increased reactive oxygen species (ROS) is one of the therapeutic effects of anticancer drugs and oxidative stress-caused mitochondria swelling is observed in resveratrol-treated cancer cells, the oxidative statuses and their relevance with resveratrol sensitivities are elucidated using THJ-16T and THJ-11T ATC cells established from two human anaplastic thyroid carcinoma cases. The results revealed that resveratrol-treated THJ-16T rather than THJ-11T cells showed remarkable growth arrest and extensive apoptosis accompanied with the elevated ROS generation and the attenuated superoxide dismutase 2 (SOD2) and catalase (CAT) levels. Mitochondrial impairment and the enhanced caspase-9/caspase-3 activation are found only in resveratrol-sensitive THJ-16T cells. Treatment with the antioxidant N-acetylcysteine (NAC) partly attenuated resveratrol-induced ROS generation and apoptosis of THJ-16T cells. The levels of resveratrol metabolic enzymes (SULT1A1 and SULT1C2) in THJ-16T cells were lower than those in THJ-11T cells and therefore reversely related with resveratrol sensitivities of ATC cells. Our findings demonstrate the ability of resveratrol to increase ROS generation and oxidative-related cellular lesions in resveratrol-sensitive THJ-16T cells presumably through activating the ROS-mitochondrial signal pathway. The levels of SULTs and ROS may reflect the response manners of ATC cells to resveratrol.

## 1. Introduction

Anaplastic occurs in less than 2% of all thyroid cancers (TCs) but accounts for about 50% of TC-related death [1, 2]. Surgery, radiotherapy, and chemotherapy and their combination are employed in ATC treatment. However, the therapeutic efficacy of those therapies is unsatisfactory and 40–60% of ATC patients died within a few months after diagnosis [3]. One major challenge to the current treatment modality for ATC is to explore a reliable therapeutic agent to suppress this extremely fast-growing and aggressive malignancy [4].

A body of evidence demonstrates that resveratrol, 3,5,4′-trihydroxystilbene, has a wide range of health benefits including chemoprevention, anti-inflammatory, antioxidant, and anticancer activities [5–8]. THJ cell lines were established in the Copland laboratory from different human anaplastic thyroid carcinoma tissues [9]. We recently found that some THJ cell lines including those with retinoic acid resistance (THJ-16T and THJ-21T) were sensitive to resveratrol in terms of distinct growth arrest and extensive apoptosis, indicating the potential therapeutic values of this nontoxic polyphenol compound in the practical treatment of ATCs [10]. However, the THJ-11T cell line had little response to resveratrol treatment due to certain unknown reason(s). It would be of clinical significance to investigate the underlying factors that influence resveratrol sensitivities of ATC cells.

Reactive oxygen species (ROS), a group of highly reactive ions and molecules, are generated in and eliminated from the cells via a variety of complex synthesis and derivative pathways and recognized as powerful signaling molecules involved in the regulation of various biological processes including the cell crisis caused by anticancer drugs [11]. Because mitochondria are the major source of cellular ROS, stimulation of mitochondrial ROS production becomes one of the anticancer strategies [12]. In cancer cells, higher ROS levels result in mitochondrial oxidative damage and the formation of mitochondrial selling which triggers apoptosis cascade by releasing apoptotic signals [13]. Redox regulation takes place via control of single enzymatic activity or at the transcriptional level [14], and its status is an important determinant of the fates of cancer cells. It is therefore proposed that the amount of ROS generation and the efficiency of its dynamic regulation may influence/determine the response manners of cancer cells to chemotherapy [15–17]. Antioxidant activity is known as one of the beneficial effects of resveratrol on normal cells, while the corresponding data from cancer cells remain lesser known [18]. Recently, we found abundant spheroid mitochondria in resveratrol-suppressed ovarian cancer cells [19]. This phenomenon indicates that resveratrol may increase rather than reduce oxidative stress in cancer cells presumably due to the poorly operated intracellular resveratrol metabolic machinery in cancer cells [20]. Given the above data, we consider that the oxidative statuses may be a possible element to determine resveratrol sensitivities of ATC cells. This study is aimed at addressing this speculation using a pair of resveratrol-sensitive and -resistant ATC cell lines.

## 2. Materials and Methods

### 2.1. Chemicals and Antibodies

Resveratrol, dimethylsulfoxide (DMSO), N-acetyl-L-cysteine (NAC), and methylthiazolyldiphenyl-tetrazolium bromide (MTT) were purchased from Sigma-Aldrich Co. (St. Louis, MO, USA). Terminal deoxynucleotidyl transferase- (TdT-) mediated dUTP nick end labeling (TUNEL) kit was purchased from Roche Inc. (Germany). MitoSOX™ Red mitochondrial superoxide indicator was purchased from Invitrogen (Molecular Probes, Invitrogen, OR, USA). 2′-7′-Dichlorodihydrofluorescein diacetate (DCFH-DA) was purchased from Beyotime Institute of Biotechnology (Jiangsu, China). The antibodies against SOD2, CAT, SULT1A1, and SULT1C2 were purchased from Proteintech (Chicago, IL, USA), and pro-caspase-3, active-caspase-3, pro-caspase-9, and active-caspase-9 from Abcam (Cambridge, UK).

### 2.2. Cell Lines and Cell Culture

The ATC cell lines THJ-16T and THJ-11T were a kind gift from Dr. Liu (Institute of Cancer Stem Cell, Dalian Medical University, as the general gifts of Mayo Foundation for Medical Education and Research). These cell lines were maintained in RPMI 1640 (GE Healthcare Life Sciences, HyClone Laboratories, Utah, USA) supplemented with 5% (for THJ-16T) or 10% (for THJ-11T) fetal bovine serum (Gibco Life Science, Grand Island, NY, USA), 100 IU/ml penicillin, and 100 *μ*g/ml streptomycin in a humidified atmosphere of 5% CO_2_ in air at 37°C. The cells (5 × 10^4^/ml) were cultured in conventional dishes (NUNC, Denmark) and seeded to high-throughput coverslip preparation dishes (Jet Biofil, Guangzhou, China; China innovation patent) to prepare sufficient cell-bearing coverslips for different experimental purposes under the same experimental condition. Resveratrol was dissolved in DMSO and added to cell culture dishes at a concentration of 100 *μ*M.

### 2.3. Cell Proliferation and Death Assays

Hematoxylin-eosin (H/E) staining was performed on cell-bearing coverslips to evaluate the morphological features of the two ATC cell lines with different treatments. The effects of resveratrol on cell proliferation were determined by 3-[4,5-dimethylthiazol-2-yl]-2,5-diphenyl-tetrazolium bromide (MTT) assay and shown in OD values. TUNEL assay was employed to detect apoptotic cells according to the producer's instructions (Promega Corporation, USA) and then photographed using a fluorescence microscope (Leica DMI4000B, Germany).

### 2.4. Annexin V-PI Staining Assay

Cells (2 × 10^5^/well) were seeded into 6-well plates. After 24 hours of incubation, before the cells were further cultured or treated with 100 *μ*M resveratrol for 48 hours and then harvested and stained with Annexin V-PI according to the protocol provided by the manufacturer (Nanjing KeyGen Biotech Co., Ltd., Nanjing, China). The labeled cells were identified by flow cytometry BD Accuri C6 (BD Biosciences, San Jose, CA, USA).

### 2.5. Ultrastructural Examination

THJ-11T and THJ-16T cells without and with 48 h 100 *μ*M resveratrol treatment were harvested, washed with PBS for three times (10 minutes/time), and fixed in 2.5% glutaraldehyde (30 min, 50 mM cacodylate buffer, pH 7.2) and 2% OsO_4_ (30 min, same buffer). Ultrathin sections (0.1 *μ*M) were prepared and examined under a Philips CM100 transmission electron microscope (FEI Company, USA). Images were captured by a charge-coupled device camera equipped with TCL-EM-Menu version 3 from Tietz Video and Image Processing Systems (Gaunting GmbH, Friedrichshafen, Germany) as described elsewhere [21].

### 2.6. Determination of Intracellular ROS Levels

2′-7′-Dichlorodihydrofluorescein diacetate (DCFH-DA), a nonfluorescent cell-permeant compound, is cleaved by intracellular esterases and then converted into the fluorescent compound upon oxidation by ROS [22]. Peroxide-dependent DCFH-DA oxidation assay (Beyotime Institute of Biotechnology, Jiangsu, China) was used to determine the intracellular ROS level. Briefly, THJ-11T and THJ-16T cells were treated with 100 *μ*M resveratrol for 0 h, 6 h, 12 h, 24 h, and 48 h. 2 × 10^5^ cells in each of time points were washed with medium and were incubated with 10 *μ*M DCFH-DA in RPMI 1640 for 20 min at 37°C in the dark. Afterward, cells were washed twice with RPMI 1640 and 1 × 10^4^ cells were analyzed using a flow cytometer (FACSCalibur, BD Biosciences, San Diego, CA, USA). In parallel, the cell coverslips were collected at each of the time points, stained in situ with DCFH-DA, and observed and photographed under a fluorescence microscope (Leica DMI4000B, Germany).

### 2.7. Determination of Mitochondrial Superoxide

MitoSOX Red (Molecular Probes, Invitrogen, Eugene, OR, USA) reacts with mitochondrial superoxide and becomes detectable fluorescent molecules under a fluorescence microscope. This experimental approach was employed to ascertain resveratrol-caused mitochondrial ROS stress according to the manufacturer's protocol. Briefly, the coverslips bearing THJ-16T cells were collected at each of observation time points, washed with DPBS twice, and then incubated with 5 *μ*M MitoSOX red for 10 minutes at 37°C. After washing with DPBS twice, the stained cells were observed and photographed under a fluorescence microscope (Leica, DMI4000B, Germany).

### 2.8. Pretreatment with ROS Inhibitor

In order to determine the involvement of ROS during resveratrol-induced toxicity to THJ-16T cells, a pretreatment with classical antioxidant N-acetylcysteine (NAC) was performed at 12 h and 48 h. Six experimental groups were set as follows: N: normal culture; NAC: treatment with 5 mM NAC; R12/R48: treatment with 100 *μ*M resveratrol for 12 h or 48 h; and NAC + R12/R48: pretreatment with 5 mM NAC for 1 h and subsequently exposed to 100 *μ*M resveratrol for 12 h or 48 h. The influence of NAC in THJ-16T cells was determined by MTT assay, DCFH-DA staining, and MitoSOX staining.

### 2.9. Western Blot Analysis

For Western blotting, total cellular proteins were prepared from the cells by the method described previously [23]. Fifty micrograms of sample protein was separated with 12% SDS/PAGE and transferred to a polyvinylidene difluoride membrane (Amersham, Buckinghamshire, UK). The membrane was blocked with 5% skimmed milk in NaCl/Tris-T (10 mM Tris/HCl, pH 8.0, 150 mM NaCl, and 0.5% Tween-20) at 4°C overnight. It was rinsed three times (10 min each) with NaCl/Tris-T, and this was followed by 2 h of incubation with the first antibodies in the appropriate concentrations (*β*-actin, 1 : 3000; SOD2, 1 : 4000; Cat, 1 : 3000; pro-caspase-9, 1 : 300; pro-caspase-3, 1 : 500; active-caspase-9, 1 : 300; active-caspase-3, 1 : 500; SULT1A1, 1: 1000; and SULT1C2, 1: 600) and 1 h of incubation with horseradish peroxidase-conjugated anti-rat IgG (Zymed Laboratories, San Francisco, CA, USA). Immunolabeling was detected with an enhanced chemiluminescence system (Roche, Mannheim, Germany) and visualized with the UVP BioSpectrum Imaging System (UVP, Upland, CA, USA). ImageJ was used to measure the density of bands (National Institutes of Health, Bethesda, MD). *β*-Actin was used as the internal quantitative control in densitometry analyses.

### 2.10. Statistical Analysis

All experiments were repeated at least three times, and the data obtained were analyzed together. The statistical significance between groups was determined with Student's *t*-test or one-way ANOVA. Data analyses were performed using SPSS software (version 17.0; SPSS, Chicago, IL). When required, *P* values are stated in the figure legends.

## 3. Results

### 3.1. Different Resveratrol Sensitivities of THJ-16T and THJ-11T Cells

**Figure 1:**
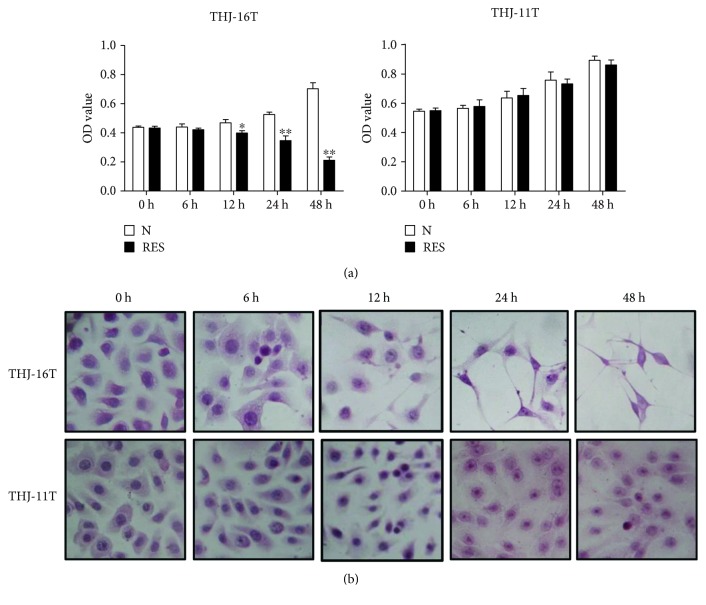
Distinct response of THJ-16T and THJ-11T cells to resveratrol. (a) MTT assay performed on THJ-16T and THJ-11T cells without (N) and with treatment with 100 *μ*M resveratrol (R) for 0, 6, 12, 24, and 48 h. Compared with the N group at the same time, ^∗^*P* < 0.05 and ^∗∗^, *P* < 0.01; (b) hematoxylin and eosin morphological staining.

The effects of resveratrol on the proliferation of two ATC cells were elucidated by MTT assay (Figure 1(a)). The data from the MTT assay indicated that resveratrol inhibited the proliferation of THJ-16T cells in a time-dependent manner (12 h, *P* < 0.05; 24 h and 48 h, *P* < 0.01). On the other hand, the OD value of the 100 *μ*M resveratrol-treated THJ-11T cell population for 48 hours remained almost unchanged in comparison with that of its untreated counterpart (*P* > 0.05). HE staining revealed cell number reduction and distinct morphological alteration of THJ-16T but not THJ-11T cells (Figure 1(b)).

### 3.2. Extensive Apoptosis of Resveratrol-Treated THJ-16T Cells

**Figure 2:**
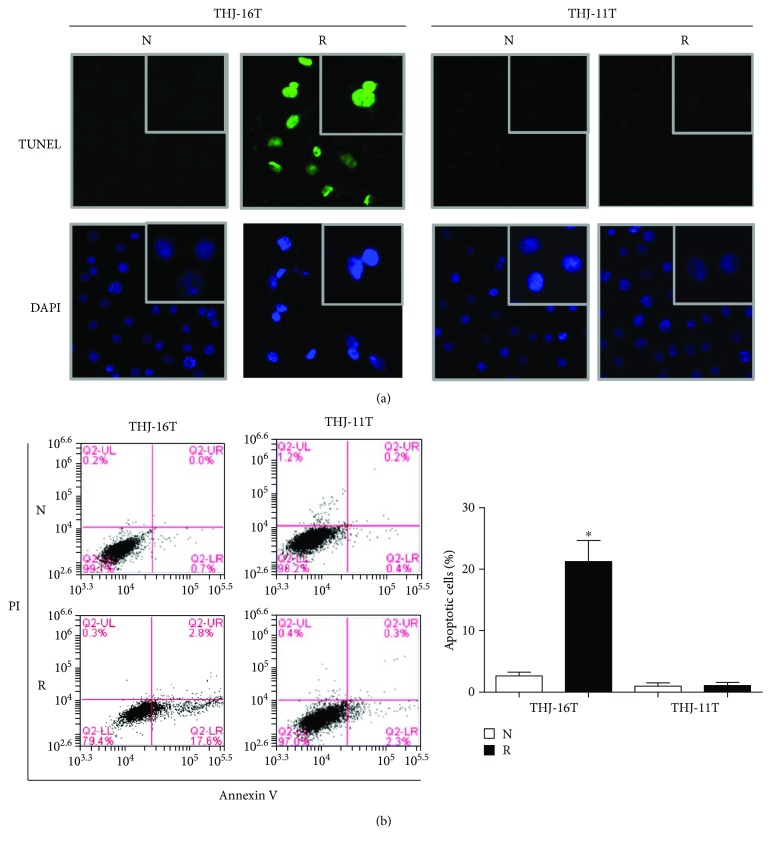
Resveratrol-induced apoptosis in THJ-16T not THJ-11T cells. (a) TUNEL staining was performed on THJ-16T and THJ-11T cells without (N) or with (R) 100 *μ*M of resveratrol for 48 h. (b) Flow cytometry analysis of Annexin V and PI for apoptosis in THJ-16T and THJ-11T cells without (N) and with (R) resveratrol treatment for 48 h. The resveratrol treatment group had significantly more cell apoptosis than the no resveratrol treatment group had in THJ-16T cells; ^∗^*P* < 0.05.

TUNEL staining demonstrated a higher percentage of nuclei with DNA damage of resveratrol-treated THJ-16T cells (Figure 2(a)). Annexin V-PI staining revealed that the proportion of apoptotic cells in normal cultured THJ-16T cells were 2.67 ± 0.49%, which increased to 21.27 ± 2.76% after a 48-hour resveratrol treatment (*P* < 0.05). In contrast, the proportion of apoptotic cells in THJ-11T cells without and with resveratrol treatment were 0.99 ± 0.42% and 1.07 ± 0.41% (*P* > 0.05; Figure 2(b)).

### 3.3. Mitochondrial Structural Alteration of Resveratrol-Treated THJ-16T Cells

**Figure 3:**
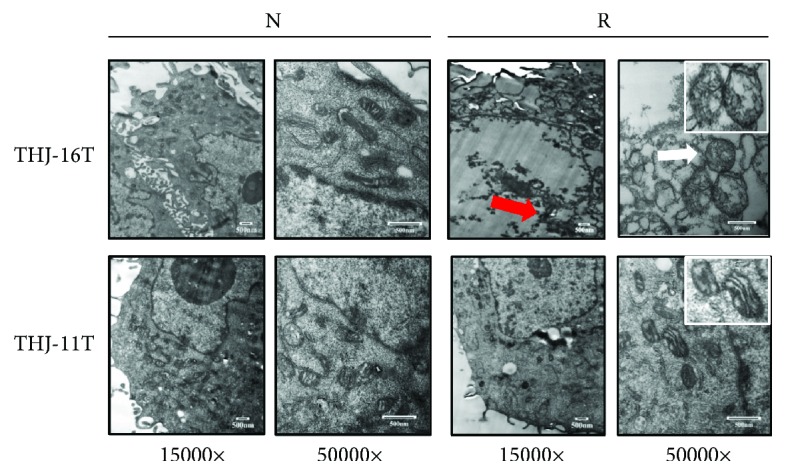
Transmission electron microscopic images of THJ-16T and THJ-11T cells without (N) and with (R) 100 *μ*M resveratrol treatment. Nucleus chromatin condensation and marginalization (red arrow), mitochondrial swelling, and mitochondria cristae breakdown (white arrow) are found in resveratrol-treated THJ-16T cells.

The ultrastructural changes of mitochondria in THJ-16T cells and THJ-11T cells without and with resveratrol treatment were examined by a transmission electron microscope. As shown in Figure 3, THJ-16T cells treated with resveratrol displayed typical apoptotic morphological changes such as chromatin condensation and fragmentation (red arrow). In comparison with the intact mitochondria of the control cells, mitochondria in the resveratrol-treated THJ-16T cells showed disappeared cristae and distinct swelling phenotype (white arrow). In contrast, the ultrastructure of THJ-11T cells as well as the mitochondria in them remained unchanged after a 48-hour resveratrol treatment.

### 3.4. Resveratrol Increased ROS Generation in THJ-16T Cells

**Figure 4:**
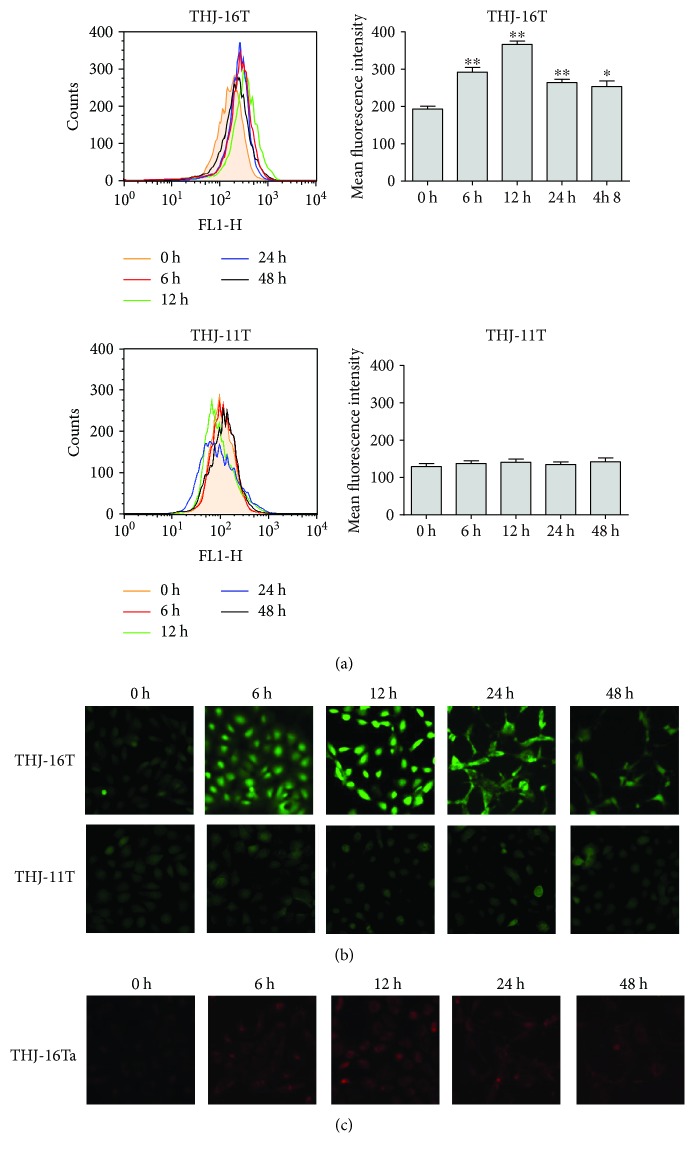
Sequential evaluation of ROS levels in THJ-16T and THJ-11T cells. The cells were treated with 100 *μ*M resveratrol for 0 h, 6 h, 12 h, 24 h, and 48 h and stained by DCFH-DA. (a) Flow cytometer determination of intracellular ROS levels in THJ-16T and THJ-11T cells. (b) Demonstration of ROS levels in THJ-16T and THJ-11T cells by fluorescence microscopy. (c) Time-course response of resveratrol-induced mitochondrial superoxide production in THJ-16T cells. All data represent the means ± SD of three independent experiments. ^∗^*P* < 0.05 and ^∗∗^*P* < 0.01 compared with those at 0 h.

It was found that ROS generation in THJ-16T cells was evaluated at 6 h, 12 h, 24 h (*P* < 0.01), and 48 h (*P* < 0.05) time points in comparison with the basal level at 0 h. In the case of THJ-11T cells, no obvious change of ROS levels was found between the cell populations without and with resveratrol treatment (*P* > 0.05; Figure 4(a)). The fluorescent microscopic findings were in accordance with flow cytometry results in terms of time-related increase of ROS levels in resveratrol-treated THJ-16T rather than THJ-11T cells (Figure 4(b)). MitoSOX staining performed on 12-hour resveratrol-treated THJ-16T cells revealed a significant increase in mitochondrial superoxide generation (Figure 4(c)).

### 3.5. Decreased SOD2 and CAT in THJ-16T Cells

**Figure 5:**
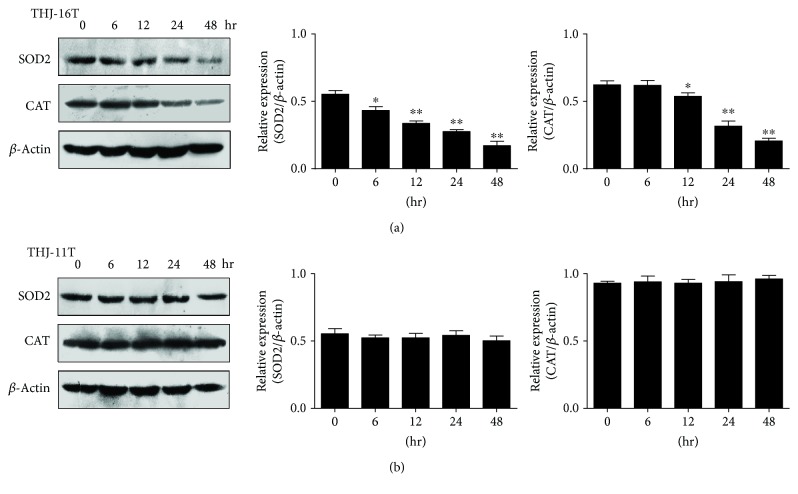
SOD2 and CAT levels in THJ-16T and THJ-11T cells. Western blot and gray density analyses of SOD2 and CAT in THJ-16T (a) and THJ-11T cells (b). *β*-Actin as the quantitative control. The statistical significance was set at ^∗^*P* < 0.05 and ^∗∗^*P* < 0.01.

To elucidate the effects of resveratrol on antioxidant enzymes, the expression of SOD2 and CAT in THJ-16T and THJ-11T cells without and with resveratrol treatment was analyzed by Western blotting (Figure 5). The results revealed that decreased levels of SOD2 and CAT were found in resveratrol-treated THJ-16T cells in a time-related manner, while no obvious reduction in SOD2 and CAT levels was observed in THJ-11T cells irrespective to resveratrol treatment.

### 3.6. Resveratrol Caused Caspase-9 and -3 Activation in THJ-16T Cells

**Figure 6:**
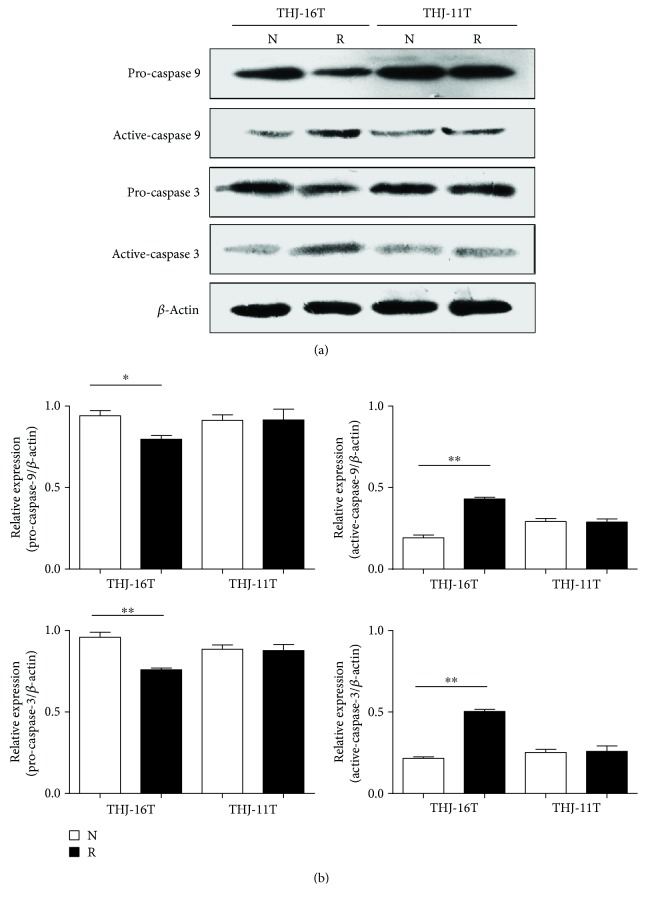
Levels of pro-caspase-9 and pro-caspase-3 and their active forms in THJ-16T and THJ-11T cells. Western blot and gray density analyses of pro-caspase-9 and pro-caspase-3 and their active form expression in THJ-16T and THJ-11T cells without (N) and with (R) 100 *μ*M resveratrol treatment. *β*-Actin as the quantitative control. The statistical significance was set at ^∗^*P* < 0.05 and ^∗∗^*P* < 0.01.

To elucidate the relevance of the mitochondria-mediated apoptotic pathway with resveratrol-induced apoptosis, the statuses of pro-caspase-9 and -3 and active-caspase-9 and -3 in THJ-16T and THJ-11T cells were checked by Western blotting (Figure 6). The levels of pro-caspase-9 and -3 were decreased by 15.3% and 20.8%; meanwhile, active-caspase-9 and -3 increased 2.3-fold and 2.4-fold after a 48-hour 100 *μ*M resveratrol treatment in comparison with those of normally cultured THJ-16T cells. The levels of the above parameters remained almost unchanged in THJ-11T cells irrespective to resveratrol treatment.

### 3.7. Downregulated SULTs in ATC Cells

**Figure 7:**
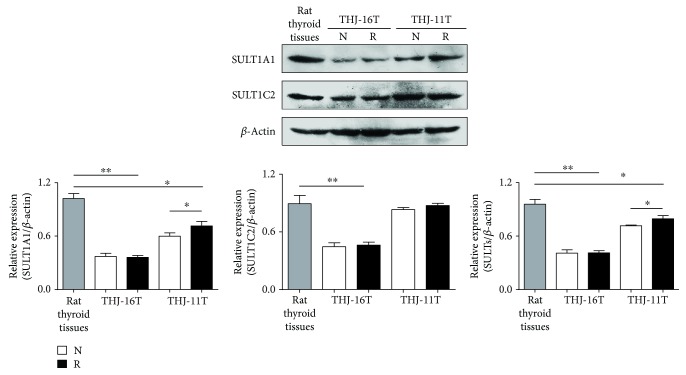
Levels of SULT1A1 and 1C2 in ATC cells without and with resveratrol treatment. Western blotting and gray density analyses of SULT1A1 and SULT1C2 expression in THJ-16T and THJ-11T cells without (N) and with (R) 100 *μ*M resveratrol treatment. *β*-Actin as the quantitative control. The statistical significance was set at ^∗^*P* < 0.05 and ^∗∗^*P* < 0.01.

Because SULTs are the major metabolic enzymes for trans-resveratrol and their levels are reversely related with chemosensitivity [19], the expression of SULT1A1 and SULT1C2 isoenzymes in the two ATC cell lines without and with resveratrol treatment was examined by Western blotting (Figure 7). The results revealed that the total SULT1A1 and SULT1C2 levels of THJ-11T cells and, especially, THJ-16T cells were 17.1% and 56.5% lower than their levels in rat normal thyroid tissues. SULT1A1 and SULT1C2 in THJ-11T cells were 16.1% and 5.0% enhanced after resveratrol treatment, while they remained unchanged in resveratrol-treat THJ-16T cells.

### 3.8. NAC Alleviated Resveratrol-Induced ROS Generation and Apoptosis in THJ-16T Cells

**Figure 8:**
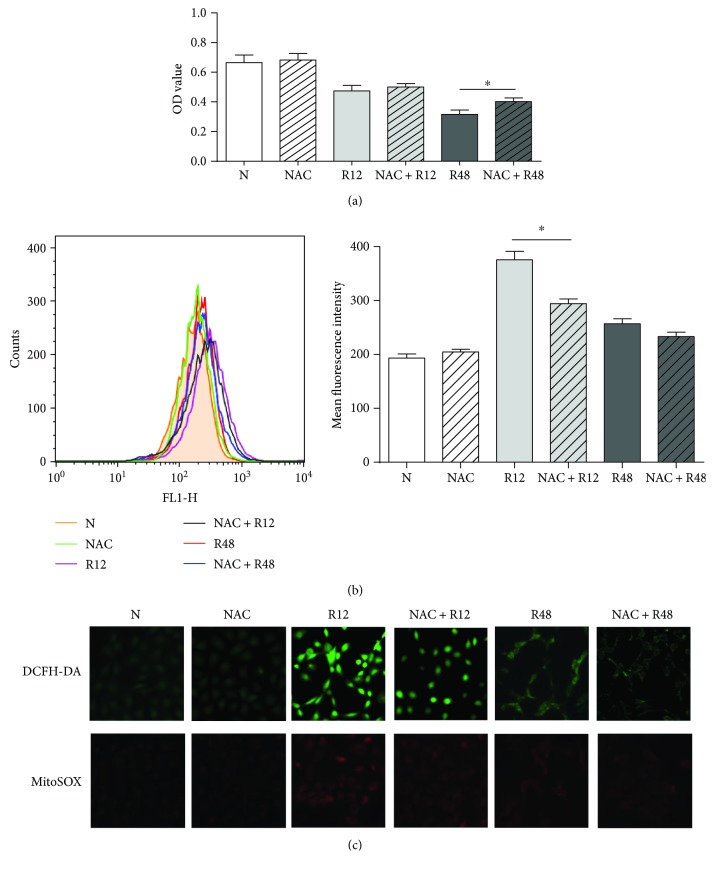
The influence of NAC pretreatment in resveratrol-induced ROS generation and cell proliferation. THJ-16T cells were pretreated with NAC (5 mM) or without NAC (5 mM) for 1 hour and then treated with or without 100 *μ*M resveratrol for 48 h. (a) MTT assay performed on THJ-16T cells of different treatment. (b) Flow cytometer determination of intracellular ROS levels in THJ-16T cells of different treatment. (c) THJ-16T cells of different treatment were stained with DCFH-DA and MitoSOX. The statistical significance was set at ^∗^*P* < 0.05. N: normal culture; NAC: treatment with 5 mM NAC; R12/R48: treatment with 100 *μ*M resveratrol for 12 h or 48 h; and NAC + R12/R48: pretreatment with 5 mM NAC for 1 h and subsequently exposed to 100 *μ*M resveratrol for 12 h or 48 h.

To further illuminate the relationship between ROS and mitochondrial signal pathways in resveratrol-induced apoptosis, THJ-16T cells were pretreated with ROS inhibitor NAC. The results of MTT assay demonstrated that the proliferation activity of the resveratrol-treated THJ-16T population was 27.2% increased by NAC at 48 h (*P* < 0.05), while it was still 39.5% and 41.1% lower than that of normally cultured cells and the cells treated by NAC alone (Figure 8(a)). NAC-pretreated cells showed decreased (21.7%) ROS generation (*P* < 0.05; Figure 8(b)) as well as DCFH-DA and MitoSOX staining (Figure 8(c)) at the 12 h time point after resveratrol treatment.

## 4. Discussion

Oxidative damage caused by intracellular ROS accumulation is one of the therapeutic effects of anticancer drugs [24, 25], and its severity is closely related with the chemosensitivities of cancer cells [26]. Oxidative damage can be reflected by mitochondrial swelling which indicates the opening of the mitochondrial permeability transition pore/MPTP and results in depolarization of mitochondrial membrane potential [27]. Resveratrol has multifacet biological activities including antioxidative and anticancer capacities [28]. These paradoxical phenomena suggest that resveratrol may exert its biological effects in cell selective patterns. For instance, resveratrol induces extensive apoptosis of human medulloblastoma cells without affecting the normal rat brain cells [29], and it inhibits the growth of ovarian cancer cells, accompanied with oxidation-related mitochondrial structural alterations [19]. It would be therefore possible that resveratrol works on normal and cancer cells in different manners and/or the molecular elements related with resveratrol action may be altered in cancer cells, resulting in different biological consequences. To elucidate these speculations, it is necessary to select resveratrol-sensitive and -insensitive cancer cells and to check their ROS levels and oxidative-related events after resveratrol treatment. As demonstrated in this study, THJ-16T and THJ-11T cells derived from highly malignant anaplastic thyroid cancers [9] are such a pair of candidates and can be used as an in vitro experimental model to address the above issues.

Anaplastic thyroid carcinoma (ATC) is the most lethal thyroid malignancy without reliable therapeutic regimen [30]. It is therefore badly in need of a novel anti-ATC agent, and resveratrol would be an option because of its nontoxic property and potential to promote cancer redifferentiation and cell death [31, 32]. This possibility is partly proved in this study, because THJ-16T cells are sensitive to resveratrol and are THJ-11T-resistant. Given the evidence of oxidation-caused mitochondrial damage in resveratrol-suppressed ovarian cancers [19], we speculated that such biological events may happen in resveratrol-treated ATC cells and are correlated with resveratrol sensitivities. Therefore, the oxidative statuses in THJ-16T and THJ-11T cells were elucidated by sequentially checking ROS generation during 48 hours of resveratrol treatment. The results showed that the ROS levels were elevated in THJ-16T cells in a time-related fashion while it remained low and stable in THJ-11T cells irrespective to resveratrol treatment. These findings demonstrated the ability of resveratrol to increase oxidative pressure in its sensitive ATC cells and the correlation of ROS level with resveratrol sensitivity. The distinct mitochondrial swelling and mitochondrial superoxide accumulation in resveratrol-treated THJ-16T rather than THJ-11T cells further support this notion, because those alterations were causally linked and lead the cells to apoptosis [33]. The partial reverse of resveratrol-caused cell crisis by NAC provides additional evidence to the suppressive effects of ROS on ATC cells and, meanwhile, suggests again the multitargeting feature of resveratrol in cancer therapy.

Cellular redox balance is typically maintained by a powerful battery of the antioxidant system in which SOD2 and CAT play active roles [11, 34]. Alternatively, reduction of those enzymes may lead to disrupted redox homeostasis, oxidative damage, and finally cell death [35]. Because of the increased ROS in resveratrol-suppressed THJ-16T but not resveratrol-resistant THJ-11T cells, the statuses of SOD2 and CAT expression in the two cell lines were analyzed in regular intervals after resveratrol treatment. It was revealed that SOD2 and CAT levels in the two ATC cell lines were similar under normal culture conditions. After drug treatment, SOD2 and CAT reduction was found only in resveratrol-sensitive THJ-16T cells in time-related patterns. This phenomenon thus indicates that the inefficiency of the antioxidant defense system in the resveratrol-treated THJ-16T cells may be responsible, at least in part, for ROS aggregation and oxidative damage [36], although the underlying mechanism of resveratrol-downregulated SOD2 and CAT expression remains to be investigated. Antioxidation is one of the beneficial effects of resveratrol on normal cells [37], while this effect is lesser mentioned in cancer cells [18]. Our results clearly demonstrate that resveratrol is able to significantly increase ROS level and, meanwhile, downregulate SOD2 and CAT production. Because these events occur only in resveratrol-sensitive ATC cells, it is reasonable to consider that the redox state of ATC cells would be one of the elements related with resveratrol sensitivity. Demonstration of oxidative cellular damage may further strengthen this notion.

It has been known that ROS accumulation is the early step involved in mitochondria-mediated apoptosis [38]. ROS overproduction causes the collapse of mitochondria, leading to the release of cytochrome C to activate caspase cascades by converting pro-caspase-9 and then pro-caspase-3 to the activated forms [39]. Therefore, the ultrastructural features, the intramitochondrial ROS level, and the statuses of caspase-3 and -9 in the two ATC cell lines without and with resveratrol treatment are investigated. The results demonstrate distinct mitochondria swelling and disappeared cristae in resveratrol-treated THJ-16T but not THJ-11T cells, which are known as the hallmarks of oxidative mitochondrial damage [27]. In accordance, the fractions of active-caspase-9 and active-caspase-3 were remarkably increased in resveratrol-treated THJ-16T cells rather than their THJ-11T counterpart. These findings provide further evidence of resveratrol-enhanced oxidative stress and its correlation with growth suppression and apoptosis of ATC cells.

It had been recognized that resveratrol suppresses cancer cell growth without affecting the corresponding normal cells [40]. Further investigation revealed that the cell-selective biological effects depend on the intracellular availability of resveratrol which has been proved to be low in normal cells because of the efficient biotransforming machinery operated by metabolic enzymes such as SULTs [20, 29, 41]. In other words, the levels of SULT expression are reversely related with resveratrol's anticancer efficacy [42]. For this reason, SULT1A2 and 1C2 were selected and their expression patterns in rat thyroid tissue and the two ATC cell lines without and with resveratrol treatment were checked. In agreement with our previous findings, these two enzymes are expressed in lower levels in THJ-11T (17.1% reduction) and, especially, in THJ-16T cells (56.5% reduction) in comparison with those in normal thyroid tissues. More importantly, this situation remains unchanged after resveratrol treatment. These findings suggest that the decreased metabolic efficiency may increase intracellular bioavailability of resveratrol in ATC cells and, as the consequence, result in distinct biological outcomes including oxidative instead of antioxidative effects. Downregulated SOD2 and CAT expression in resveratrol-treated THJ-16T ATC cells can aggravate the situation.

## 5. Conclusions

Taken together, the results of the current study demonstrate extensive apoptosis in resveratrol-treated THJ-16T cells, accompanied with a remarkably increased ROS level, mitochondrial swelling, and caspase-9 and caspase-3 activation. However, those events are not found in resveratrol-resistant THJ-11T cells under the same experimental condition. Furthermore, antioxidant reagent NAC reduces ROS accumulation and partly improves the resveratrol-caused cell crisis of THJ-16T cells. The levels of SOD2 and CAT, the major antioxidative enzymes, are reduced by resveratrol in THJ-16T but not in THJ-11T cells, indicating the decreased redox efficiency in resveratrol-sensitive ATC cells. Meanwhile, the overall levels of the major resveratrol metabolic enzymes SULT 1A1 and 1C2 are reduced in THJ-16T cells in the extent of 64.6% and 48.3% in comparison with those in rat normal thyroid tissues and THJ-11T cells, respectively, suggesting the reduced biotransforming efficiency and, alternatively, the increased bioavailability of resveratrol in resveratrol-sensitive ATC cells. Our above findings thus demonstrate (1) that resveratrol is able to cause oxidative stress in cancer cells, (2) that the increased ROS generation and oxidative damages are closely related with the suppressive effects of resveratrol on ATC cells, and (3) that SOD2 and CAT reduction and SULT downregulation may be responsible for ROS accumulation in resveratrol-sensitive ATC cells. Consequently, the levels of ROS, SOD2, CAT, and SULTs would be a group of closely related parameters to predict the therapeutic outcomes of resveratrol-based cancer therapy.

## Data Availability

All data used to support the findings of this study are included within the article.
